# Psychometric properties and factor structure of an Ecuadorian version of the Alcohol Use Disorders Identification Test (AUDIT) in college students

**DOI:** 10.1371/journal.pone.0219618

**Published:** 2019-07-10

**Authors:** Víctor López, Belén Paladines, Silvia Vaca, Raúl Cacho, Javier Fernández-Montalvo, Pablo Ruisoto

**Affiliations:** 1 Department of Psychology, Technical Particular University of Loja, Loja, Ecuador; 2 Department of Health Sciences, Public University of Navarra, Pamplona, Spain; 3 Faculty of Psychology, University of Salamanca, Salamanca, Spain; Ulster University, UNITED KINGDOM

## Abstract

**Background:**

The Alcohol Use Disorders Identification Test (AUDIT) is the gold standard in assessing harmful alcohol intake, which is responsible for substantial morbidity and mortality.

**Objective:**

The goal of this study is to evaluate the psychometric properties and factor structure of an Ecuadorian adaptation of a Spanish translation of the AUDIT in a large sample of college students in Ecuador.

**Methods:**

A total of 7905 students, including 46.26% males, and 53.75% females, from 11 universities in Ecuador, were surveyed. The questionnaire was tested for two- and three-factor structures, reliability, and correlations with other health related measures.

**Results:**

The Kaiser-Meyer-Olkin test for sampling adequacy was satisfactory (.0885), and Bartlett´s test for sphericity was significant (p < .001). Although both models showed a good fit to the data, the two-factor model was preferred based on the high correlations between the factors 2 and 3 within the three-factor model (.86 for the total sample, .77 for females, and .91 for males). The reliability for the two-factor model was good, as indicated by Cronbach´s α = .806 (factor I) and .716 (factor II) for the total sample, .808 (factor I) and .667 (factor II) for females, and .787 (factor I) and .728 (factor II) for males. Additionally, the AUDIT scores positively correlated with several health-related measures: stress, psychological inflexibility, loneliness and depression/anxiety symptomatology.

**Conclusion:**

The Ecuadorian adaptation of the Spanish version of the AUDIT has good reliability, and internal consistency and correlates with other health related measures, proving to be a reliable tool that can be used by researchers and clinicians to screen hazardous alcohol intake in college students.

## Introduction

Harmful use of alcohol causes substantial morbidity and mortality, including depression and anxiety symptoms, cognitive deficits, the abuse of other drugs, heart disease, cancer, road traffic accidents and violence, including suicide [1–2] with significant differences according to sex [3]. As a consequence, the reduction in the harmful consumption of alcohol is currently one of the major public health challenges in the western world [1,4,5,6].

The World Health Organization encourages the development and use of standardized instruments to enable cross-national comparisons. As such, the Alcohol Use Disorders Identification Test (AUDIT) was developed from a six-country WHO collaborative project as an instrument for the screening or early detection of hazardous and harmful alcohol use [7–8]. The original English-language version as well as many translated and language-adapted versions of the AUDIT are currently the gold standard a wide range of cross-national studies of both clinical samples [9–20] and the general population. College students represent an especially vulnerable population due to the underdevelopment of their prefrontal cortex and executive functions in comparison with those of adults [21–26].

The original version of the AUDIT developed by the World Health Organization originally consisted of three factors: alcohol consumption (items 1–3), drinking behavior (items 4–6), and alcohol-related problems (items 7–10). However, other studies reported a different factor structure [27–28]. For example, Bergman and Källmén [29] used a two-factor structure in a Swedish sample: “hazardous consumption” factor (items 1–3) and an “alcohol-related problems” factor (items 4–10). Shevlin & Smith [30] also employed a two-factor model in a large sample in Great Britain. Moreover, the use of two-factor models has been reported for both males and females in previous studies [27, 31, 32].

Ecuador is one of the countries with the highest rate of alcohol consumption in Latin America, according to the latest data from the World Health Organization [2]. However, an adaptation of the Spanish version of the AUDIT has yet to be developed. This work will address this gap in the currently available instruments to measure harmful alcohol consumption in the Ecuadorian population.

The aim of this study is to evaluate the psychometric properties and factor structure of an Ecuadorian adaptation of the Spanish version of the AUDIT in a large sample of college students, exploring potential differences by sex.

## Methods

### Participants

College students enrolled in 11 universities in Ecuador were invited via email to participate in the study, and they then completed a computerized survey within the 3-week assessment period. A total of 7905 participants met the inclusion criteria of being enrolled in, at least, one whole academic year; and completing the entire survey (the average response rate across universities was 47,8%, ranging from 39.10% to 56.30%). The average age was 21.49 years (SD = 3.68). Among the participants, 3656 (46.26%) were males, with an average age of 21.8 (SD = 3.7), and 4249 (53.75%) were females, with an average age of 21.2 (SD = 3.7).

### Design

A descriptive cross-sectional study was conducted. Data were collected during the first three weeks of November 2015. Participation was confidential, and fully anonymous, and a brief summary of individual scores was freely provided after the completion of the survey to encourage honest answers and a higher response rate.

The study was approved by the Ethics Committee for Research in Human Beings (*Comité de Ética de Investigación en Seres Humanos*, CEISH) of the Ministry of Public Health of the Republic of Ecuador in June 2015 (MSP-DIS-2015-0088-O) and was conducted according to the principles expressed in the Declaration of Helsinki. Written informed consent was obtained from all participants. A language adaptation of the Spanish version of the AUDIT into Ecuadorian was carried out by a panel of three experts (see S1 Appendix). The final Ecuadorian Spanish adaptation was tested on 30 candidates as a pilot trial to test for the clarity and comprehensiveness of the questionnaire.

### Measures

Alcohol Use Disorders Identification Test (AUDIT, Self-report version) [33]. The AUDIT consists of a 10-item questionnaire designed to screen for hazardous alcohol intake. Subjects respond to each question by indicating the frequency of alcohol consumption and/or the experience of symptoms related to problematic drinking on a scale of 0 ("never"), 4 ("4 or more times a week"), yielding a maximum possible score of 40. Higher scores indicate a higher risk of problematic alcohol consumption and a score of 8 or above is considered as a cut-off point for a hazardous score [34–35].

Perceived Stress Scale (PSS-14) [36]. This scale consists of 14 items to assess the degree to which people perceive a lack of control in their daily lives. Participants respond to a 5-point Likert-type scale ranging from 0 (“never”) to 4 (“very often”). Scores range from 0 to 56 points. Higher scores indicate higher levels of psychological stress. The Cronbach’s α coefficient for internal consistency reliability was .82 for males and .83 for females.

Loneliness Scale Revised-Short (UCLA) [37]. This instrument consists of a brief 3-item scale evaluating the subjective feeling of loneliness, understood as the perception of less social support being available than desired. Participants respond based on their agreement with a series of statements, where 1 = “hardly ever”, 2 = “sometimes”, and 3 = “often”. Scores range from 0 to 9. Higher scores indicate a greater feeling of loneliness or lack of social support. The Cronbach’s α coefficient for internal consistency reliability was .76 for males and .84 for females.

Acceptance and Action Questionnaire (AAQ-7) [38]. This questionnaire is the most widely used general measure of psychological inflexibility, defined as rigidity in the handling of emotions or unpleasant internal events. It consists of 7 items and participants respond to a 7-point Likert-type scale, from 1 = “never” to 7 = “always”. Scores range from 7 to 49. Higher scores indicate a tendency to act under the need to control or avoid aversive thoughts, memories or feelings. The Cronbach’s α coefficient for internal consistency reliability was .93 for males and .95 females.

Patient Health Questionnaire of Depression and Anxiety (PHQ-4) [39]. This questionnaire assesses depression and anxiety associated with functional impairment and disability days. Scores range from 0 to 12. A higher score indicates a greater anxiety and depression level.

### Data analysis

The statistical analyses were carried out using the IBM Statistical Package for the Social Sciences (SPSS) software and AMOS version 21.0 for Windows. All analyses were carried out separately for the total, male and female samples (see S1 Database). Considering the skewed distribution of AUDIT scores, descriptive analyses were carried out using the median and interquartile range (IQR). Mann-Whitney U test was conducted as a non-parametric equivalent to the independent group test to compare differences between males and females. Then, a confirmatory factor analysis was conducted to reveal the factor structure of the AUDIT. The Kaiser-Meyer-Olkin test was used to assess sampling adequacy, and Bartlett´s test was used to assess sphericity assumptions. To assess the overall fit of the factor model, the following indexes were calculated: chi squared (χ^2^), comparative fit index (CFI), Tucker Lewis Index (TLI), root mean square error of approximation (RMSEA), standardized root mean square residual (SRMR) and Akaike information criterion (AIC). Cronbach´s α was used to assess the reliability for the global score of two- and three-factor solutions in men, women and the total sample. In addition, Cronbach´s α was calculated independently for each item is displayed independently. Finally, two-tailed Pearson's correlations of the AUDIT scores with other health-related measures were conducted.

## Results

### AUDIT scores

Total scores, and scores by item are displayed in Table 1 for the total, male and female samples independently. Males scored significantly higher than females on the total scale and each one of the items. In fact, 46.74% of the male college students (n = 1709), and 24.14% of the females students in the sample (n = 1026), reported a score above the cut-off of 8 points (AUDIT score ≥ 8) for harmful alcohol consumption, and the 34.59% (n = 2735) of the total sample reported a score above the cutoff.

**Table 1:** Audit scores in in the total sample and differences between females and males.

	Total sample (N = 7905)	Females (n = 4249)	Males (n = 3656)		
Items	Median (IQR)	Median (IQR)	Median (IQR)	*U*	*p*
1. How often do you have a drink containing alcohol?	1 (2–1)	1 (1–0)	1 (2–1)	6111278,5	< .001
2. How many drinks containing alcohol do you have on a typical day when you are drinking?	1 (2–0)	1 (2–0)	2 (3–0)	5613820,0	< .001
3. How often do you have six or more drinks on one occasion?	1 (1–0)	1 (1–0)	1 (2–1)	5914701,5	< .001
4. How often during the last year have you found that you were not able to stop drinking once you had started?	0 (1–0)	0 (0–0)	0 (1–0)	6276310,0	< .001
5. How often during the last year have you failed to do what was normally expected from you because of drinking?	0 (0–0)	0 (0–0)	0 (1–0)	6738524,5	< .001
6. How often during the last year have you needed a first drink in the morning to get yourself going after a heavy drinking session?	0 (0–0)	0 (0–0)	0 (0–0)	7157075,0	< .001
7. How often during the last year have you had a feeling of guilt or remorse after drinking?	0 (1–0)	0 (1–0)	0 (1–0)	6668464,5	< .001
8. How often during the last year have you been unable to remember what happened the night before because you had been drinking?	0 (1–0)	0 (0–0)	0 (1–0)	6529972,5	< .001
9. Have you or someone else been injured as a result of your drinking?	0 (0–0)	0 (0–0)	0 (0–0)	7292890,0	< .001
10. Has a relative or friend or a doctor or another health worker been concerned about your drinking or suggested you cut down?	0 (0–0)	0 (0–0)	0 (0–0)	6940759,5	< .001
Total score	5 (9–2)	4 (7–0)	7 (11–3)	5415375,0	< .001

### Factor structure

To determine whether the sample was large enough to perform a factor analysis, the Kaiser-Meyer-Olkin (KMO) measure was analyzed, yielding a KMO value = .885 for the total sample (.865 for females and .883 for males) indicating that the sampling was close to optimal. In addition, the assumption of sphericity was met as indicated by Bartlett's sphericity test: χ^2^(45) = 25138.625, *p* < .001 for the total sample; χ^2^(45) = 12179.569, *p* < .001 for females and χ^2^(45) = 11388.985, p < .001 for males. The confirmatory factor analysis performed by sex suggested that the questionnaire performed equally well for males and females with both the 2 and three-factor solutions (Table 2).

**Table 2:** Distribution of Items factor load of the items for the two- and three-factor solutions for the total, male and female samples.

	2 factors						3 factors								
	Total sample (N = 7905)		Females (n = 4249)		Males (n = 3656)		Total sample (N = 7905)			Females (n = 4249)			Males (n = 3656)		
Items	1	2	1	2	1	2	1	2	3	1	2	3	1	2	3
1	.83		.83		.81		.83			.83			.81		
2	.71		.70		.68		.71			.70			.68		
3	.91		.90		.92		.91			.90			.92		
4		.62		.59		.61		.66			.66			.64	
5		.67		.64		.68		.73			.73			.72	
6		.45		.37		.48		.47			.39			.49	
7		.66		.64		.66			.68			.68			.67
8		.58		.57		.57			.60			.59			.58
9		.36		.29		.40			.37			.30			.41
10		.48		.44		.49			.50			.45			.50
Factor correlations															
Factor 1	1.0		1.0		1.0		1.0			1.0			1.0		
Factor 2	.74	1.0	.73	1.0	.73	1.0	.67	1.0		.62	1.0		.67	1.0	
Factor 3							.75	.86	1.0	.72	.77	1.0	.75	.91	1.0

The confirmatory factor analysis reported goodness of fit indexes for both models: the 2-factor and 3-factor solutions. The AIC index was also low for both solutions (2-factors AIC = 532.6; 3-factors AIC = 303.1). Therefore, based on the logic of parsimony, the 2-factor solution is preferred, with the first factor corresponding to items 1–3, the second factor corresponding to items 3–10 (Table 3).

**Table 3:** Goodness of fit indexes for the confirmatory factor analysis.

	2 factors				3 factors	
	Total sample (N = 7905)	Females (n = 4249)	Males (n = 3656)	Total sample (N = 7905)	Females (n = 4249)	Males (n = 3656)
X^2^(*df*)	490.6 (*34*)	380.1 (*34*)	239.8 (*34*)	257.1 (*34*)	131.9 (*34*)	178.8 (*34*)
RMSEA	.041	.049	.041	.030	.027	.035
(90% CI)	(.038-.044)	(.045-.053)	(.036-.046)	(.027-.033)	(.022-.032)	(.030-.041)
SRMR	.0238	.0284	.0250	.0175	.0182	.0212
CFI	.982	.972	.982	.991	.992	.987
TLI	.976	.962	.976	.987	.989	.982
AIC	532.6	442.1	301.8	303.1	197.2	244.8

Root mean square error of approximation (RMSEA); Standardized root mean square residual (SRMR); Comparative fit index (CFI); Tucker-Lewis index (TLI); Akaike information criterion (AIC).

### Reliability

The Ecuadorian adaptation of the Spanish AUDIT scale was found to be highly reliable for the total sample (*α* = .818), for females *α* = .795, and males (*α* = .816). Table 4 displays the matrix of Cronbach *α* coefficients for each item with the total score and *α* coefficients when each item is removed. Items 6, 9 and 10 failed to reach α coefficients above .50 for the total, female and male samples. Item 4 was also slightly below the expected .50 value. The reliability for the two-factor model was superior to that of the three-factor model for the total, female and male samples (Table 4).

**Table 4:** Internal consistency for AUDIT total scores in the two- and three-factor models tested. Th subscales and items for the total, female and male samples are displayed.

Crombach’s *α*	Global scale	2-factor model		3-factor model		
		Factor 1 (Items 1–3)	Factor 2 (Items 4–10)	Factor 1 (Items 1–3)	Factor 2 (Items 4–6)	Factor 3 (Items 7–10)
**Total sample** (N = 7905)	.818	.806	.716	.806	.615	.592
**Females** (n = 4249)	.795	.808	.667	.808	.579	.539
**Males** (n = 3656)	.816	.787	.728	.787	.620	.603
	**Total sample (N = 7905)**		**Females (n = 4249)**		**Males (n = 3656)**	
	Item—test correlation	α if item removed	Item—test correlation	α if item removed	Item—test correlation	α if item removed
Item 1	.675	.785*	.672	.754*	.654	.786*
Item 2	.576	.801**	.568	.772*	.535	.803**
Item 3	.734	.778*	.711	.749*	.727	.777*
Item 4	.516	.800*	.472	.777*	.515	.798*
Item 5	.551	.800*	.515	.776*	.556	.797*
Item 6	.380	.816**	.309	.796*	.398	.812**
Item 7	.566	.796*	.548	.770*	.556	.794*
Item 8	.506	.802**	.470	.778*	.502	.799*
Item 9	.331	.822**	.270	.806**	.367	.816**
Item 10	.431	.812**	.379	.790*	.436	.811**

Internal consistency

*acceptable (.8 > α > .7)

**good (.9 > α > .8).

### AUDIT correlations with other health-related measures

Correlations between the AUDIT scores and 4 well-established health related measures in the scientific community were statistically significant: stress (r = .156, *p* < .001 in males and r = .157, *p* < .001 in females), loneliness (r = .114, *p* < .001 in males and r = .150, *p* < .001 in females), psychological inflexibility (r = .176, *p* < .001 in males and r = .213, *p* < .001 in females), and depressive and anxiety symptoms (r = .169, *p* < .001 in males and r = .171, *p* < .001 in females).

## Discussion

A linguistically adapted and culturally acceptable equivalent of the Spanish version of the Alcohol Use Disorders Identification Test (AUDIT) was tested among a large sample of college students in Ecuador, reporting good reliability and a factor structure consistent with the original version.

As far as we know, this is the first and largest study to assess the appropriateness, meaningfulness, and usefulness of an adaptation of the AUDIT for measuring the risk of harmful alcohol consumption in college students in Ecuador; thus, this study responds to the need to explore psychometric properties in non-English versions and translated adaptations demanded by Reinert and Allen [40–41].

Sex differences deserve special attention. Males reported significantly higher levels of alcohol consumption than females. The score for the total sample was almost 2 points below the cut-off point of 8 assumed for harmful alcohol consumption. However, it is worth noting that alcohol abstainers were included in the calculation of that value, which explains the 5-point standard deviation. Furthermore, the prevalence of harmful alcohol consumption, above the cut-off score of 8, was strikingly high. Almost 50% of male college students and 25% female college students reported harmful alcohol consumption. Previous studies have reported a significantly higher risk for harmful drinking (AUDIT score > 8) in college students than in noncollege youth [42] and differences across countries [43]. The rates of harmful alcohol consumption (AUDIT score > 8) reported by college students in this study are similar to the rates reported in North America or New Zealand (countries with the highest rates, above 50% for males and females) [43], higher than Europe and South America (where rates range between 23–33% for males and 10–22% for females)[43], Africa, Asia, and counties from the Arab region (with the lowest reported rates, below 10%) [43]. In comparison with Ecuador, the rates of harmful alcohol consumption (AUDIT score > 8) found in this study are similar to the 39% prevalence of abusive drinking reported almost 15 years ago in the first research conducted in a college sample in Ecuador [44].

Sex differences are consistent with previous studies, which have already found both a high prevalence of harmful consumption among the general population and higher rates in males than in females [2,5]. The aforementioned pattern of sex differences was found not only in the AUDIT total scores but also in the scores for each item. Different scores between males and females may either support the use of different cut-off scores or evidence lower levels of harmful drinking in women [45]. Previous studies consider that women are more sensitive to alcohol than men, recommending lower the cut-off score for women from the recommended value of 8 points to a value of 6 points [27, 29, 40].

Regarding the reliability of the AUDIT, our results yielded a Cronbach's α = .818 for the total scale, .816 for males, and .795 for females, indicating the good reliability of the Ecuadorian adaptation of the Spanish version of the AUDIT. These results are in line with previous studies, which generally have reported Cronbach's α and item-total correlations values of approximately 0.80 [46].

Regarding the factor structure, confirmatory factor analysis supported the two-factor model and the three-factor models consistent with the original conceptual domains [28]. Indeed, the goodness-of-fit indexes provided support for both, the two and three-factor models, with both fitting the data equally well. However, the two-factor model is preferred in Ecuador based on the principle of parsimony, given the very high correlations between the factors 2 and 3 in the three-factor model, in accordance with previous studies aimed at detecting patients at risk for problematic drinking behavior in primary care [27,29,30,31]. It is worth noting that the factor loadings for item 9 were very low for both models, especially in the two factor models for females (0.29). Moreover, the correlation of item 9 with the total score was the lowest for the total sample (.33), females (.27) and males (.36). The poor functioning of this item has already been reported previously by the literature [32] suggesting the exclusion of this item because it does not generate score variability.

In addition, the AUDIT scores positively correlated with multiple well-established health-related measures, including psychological stress [36,43], loneliness [37], psychological inflexibility [38] and depressive/anxiety symptoms [39]. See Cohen, Janicki-Deverts, & Miller [47] or Remor [36] for a detailed description of the impact of stress on health; Cacioppo [48] or Hughes, et al. [37] for a review of the impact of loneliness on health; Kashdan & Rottenberg [49] or Bond et al. [38] for the role of psychological inflexibility on health; and Wingenfeld, et al. [39] for the link between depressive and anxiety symptoms and health. Moreover, although we acknowledge that correlations between AUDIT scores and other health outcomes measures of alcohol do not provide evidence about the validity of the AUDIT, such correlations have been used by previous studies to highlight the health implications of alcohol consumption for other health dimensions [32]. Future studies should explore further the validity of the AUDIT against other measures of alcohol use in college students in Ecuador.

The remarkably high AUDIT scores in our sample, especially among males, might be useful for anticipating and raising concerns about expected adverse health outcomes in this population in the near future. Other studies have already used AUDIT scores to predict alcohol-related illness, social problems, hospital admission [35], the development of alcohol withdrawal symptoms during inpatient detoxification [50], and even mortality over a 2-3-year period [35].

Finally, our results are consistent with previous studies that have reported the good psychometric properties of the English version of the AUDIT and other versions. Future studies should further explore the following areas: 1) the psychometric properties and factor structure of short versions of the AUDIT, for example, AUDIT-C, the most popular short version, which consists of the 3 consumption items of the AUDIT, and which seems to work as well as the full AUDIT [40,41,51,52]; 2) the correlation between AUDIT scores and biomarkers of alcohol drug abuse, including measures of liver enzymes, blood volume, alcohol byproducts, such as acetaldehyde, or differences in beta-endorphin or gamma-aminobutryic acid [53], in order to identify harmful cut-off scores for males and females in nonclinical samples [9]; and 3) the potential role of the item sequence should also be explored more systematically [54].

## Supporting information

S1 Appendix Ecuadorian-Spanish version of AUDIT. (DOCX)

S1 Database Set of data. (ZIP)
