# Supplementary material for: Psychometric properties and factor structure of an Ecuadorian version of the Alcohol Use Disorders Identification Test (AUDIT) in college students
Source: PLoS One. 2019 Jul 10;14(7):e0219618. doi: 10.1371/journal.pone.0219618 (PMC6619822; doi:10.1371/journal.pone.0219618)
Supplement: S1 Appendix — (DOCX) [file pone.0219618.s001.docx]

**Appendix**

Ecuadorian-Spanish version of AUDIT

| Selecciona la opción que mejor describa su respuesta a cada pregunta. | | | | | |
| --- | --- | --- | --- | --- | --- |
| 1. ¿Con qué frecuencia consume alguna bebida alcohólica? Ej. (cerveza, cocktails, etc.) | (0)  Nunca | (1)  Una o menos veces al mes | (2)  De 2 a 4 veces al mes | (3)  De 2 a 3 veces a la semana | (4)  4 o más veces a la semana |
| 2. ¿Cuantas bebidas alcohólicas suele tomar en un día normal cuando bebe? | (0)  1 o 2 | (1)  3 o 4 | (2)  5 o 6 | (3)  7, 8, o 9 | (3)  10 o más |
| 3. ¿Con qué frecuencia toma 6 o más bebidas alcohólicas en un solo día? | (0)  Nunca | (1)  Menos de una vez al mes | (2)  Mensualmente | (3) Semanalmente | (4)  A diario o casi a diario |
| 4. En el último año ¿Con qué frecuencia ha sido incapaz de parar de beber una vez había  empezado? | (0)  Nunca | (1)  Menos de una vez al mes | (2)  Mensualmente | (3) Semanalmente | (4)  A diario o casi a diario |
| 5. En el último año ¿Con qué frecuencia no pudo hacer lo que se esperaba de usted porque había bebido? | (0)  Nunca | (1) Menos de una vez al mes | (2)  Mensualmente | (3) Semanalmente | (4)  A diario o casi a diario |
| 6. En el último año ¿Con qué frecuencia ha necesitado beber al levantarse para recuperarse después de haber bebido mucho el día anterior? | (0)  Nunca | (1)  Menos de una vez al mes | (2)  Mensualmente | (3) Semanalmente | (4)  A diario o casi a diario |
| 7. En el último año ¿Con qué frecuencia ha tenido remordimientos o se ha sentido culpable después de haber bebido? | (0) Nunca | (1)  Menos de una vez al mes | (2)  Mensualmente | (3) Semanalmente | (4)  A diario o casi a diario |
| 8. En el último año ¿Con qué frecuencia no ha podido recordar lo que sucedió la noche anterior porque había estado bebiendo? | (0) Nunca | (1)  Menos de una vez al mes | (2)  Mensualmente | (3) Semanalmente | (4)  A diario o casi a diario |
| 9. ¿Usted o alguna otra persona ha resultado herido porque usted había bebido? | (0)  No |  | (2)  Sí, pero no en el curso del último año |  | (4)  Sí, el último año |
| 10. ¿A algún familiar, amigo, médico o profesional sanitario ha preocupado su consumo de bebidas alcohólicas o le ha sugerido que beba menos? | (0)  No |  | (2) Sí, pero no en el curso del último año |  | (4) Sí, el último año. |

Selection of response (0) in ítem 1 automatically led to item 9 and 10. If summation of items 2 and 3 = 0, then participant was led to items 9 and 10
